# Ozone exposure, vitamin C intake, and genetic susceptibility of asthmatic children in Mexico City: a cohort study

**DOI:** 10.1186/1465-9921-14-14

**Published:** 2013-02-04

**Authors:** Hortensia Moreno-Macías, Douglas W Dockery, Joel Schwartz, Diane R Gold, Nan M Laird, Juan J Sienra-Monge, Blanca E Del Río-Navarro, Matiana Ramírez-Aguilar, Albino Barraza-Villarreal, Huiling Li, Stephanie J London, Isabelle Romieu

**Affiliations:** 1 Universidad Autónoma Metropolitana, Unidad Iztapalapa, Avenida San Rafael Atlixco 186, edificio H-001, Col. Vicentina, 09430, D F, México City, Mexico; 2 Environmental Health Department, Harvard School of Public Health, Boston, MA, USA; 3 Biostatistics Department, Harvard School of Public Health, Boston, MA, USA; 4 Hospital Infantil “Federico Gómez”, México City, Mexico; 5 Comisión Federal para la Protección contra Riesgos Sanitarios, SSA, México City, Mexico; 6 Instituto Nacional de Salud Pública, Cuernavaca, Morelos, Mexico; 7 U.S. Department of Health and Human Services, Epidemiology Branch, National Institute of Environmental Health Sciences, National Institutes of Health, , USA; 8 International Agency for Research on Cancer, Lyon, France

**Keywords:** Air pollution, Asthmatic children, Antioxidant genes, Mexico City, Vitamin C

## Abstract

**Background:**

We previously reported that asthmatic children with *GSTM1* null genotype may be more susceptible to the acute effect of ozone on the small airways and might benefit from antioxidant supplementation. This study aims to assess the acute effect of ozone on lung function (FEF_25-75_) in asthmatic children according to dietary intake of vitamin C and the number of putative risk alleles in three antioxidant genes: *GSTM1*, *GSTP1* (rs1695), and *NQO1* (rs1800566).

**Methods:**

257 asthmatic children from two cohort studies conducted in Mexico City were included. Stratified linear mixed models with random intercepts and random slopes on ozone were used. Potential confounding by ethnicity was assessed. Analyses were conducted under single gene and genotype score approaches.

**Results:**

The change in FEF_25-75_ per interquartile range (60 ppb) of ozone in persistent asthmatic children with low vitamin C intake and *GSTM1* null was −91.2 ml/s (p = 0.06). Persistent asthmatic children with 4 to 6 risk alleles and low vitamin C intake showed an average decrement in FEF_25-75_ of 97.2 ml/s per 60 ppb of ozone (p = 0.03). In contrast in children with 1 to 3 risk alleles, acute effects of ozone on FEF_25-75_ did not differ by vitamin C intake.

**Conclusions:**

Our results provide further evidence that asthmatic children predicted to have compromised antioxidant defense by virtue of genetic susceptibility combined with deficient antioxidant intake may be at increased risk of adverse effects of ozone on pulmonary function.

## Background

Ozone is a potent oxidant that easily reaches the lung lining fluid compartment [1] providing an additional^a^ source of reactive oxygen species and causing, among other outcomes, reversible declines in lung function [2]. Vitamin C is an exogenous nonenzymatic antioxidant that participates in the primary lung defense against reactive oxygen species [3]. Several epidemiological studies have suggested that a diet rich in antioxidants may lessen the effect of ozone on respiratory health of susceptible populations [4,5].

With respect to genetics, epidemiological studies have shown that the effect of ozone on the respiratory health of asthmatic children may be modulated by the functional genetic polymorphisms of antioxidant genes such as *Glutathione Stransferase* (*GST*) *M1*, (*GST*)*P1*, and *NQO1*[6,7]. These genes code for phase II xenobiotic detoxifying enzymes that participate in the intracellular defense against oxidative stress. They are members of the Nrf2 pathway, responsible for the expression of enzymes that conjugate and inactivate reactive oxygen species [8].

In a randomized controlled trial of antioxidant supplementation (vitamin C and E) among asthmatic children in Mexico City, we reported that children in the placebo group with *GSTM1* null genotype may be more vulnerable to the adverse effects of ozone on lung function than those who were taking antioxidant supplementation and those with *GSTM1* positive genotype, regardless of the treatment group [9]. In the previous analysis, *GSTM1* was classified as null versus positive. In the current analysis, the number of copies of *GSTM1* (0, 1 or 2) was quantified; and we examined whether dietary intake of vitamin C in combination with common functional variants in three antioxidant genes (*GSTM1*, *GSTP1* and *NQO1*) influences the relationship between ozone exposure and lung function (FEF_25-75_) in Mexican asthmatic children exposed to high ozone levels. In addition to examining the functional polymorphisms in these three antioxidant genes separately, we created a putative antioxidant deficiency genetic risk score by combining genotypes across them.

## Methods and materials

See Additional file 1 for further explanation of the methods mentioned below.

### Study subjects

**Figure 1:**
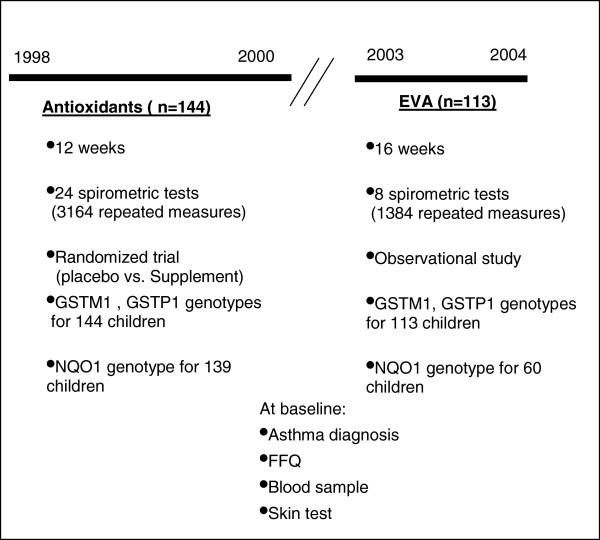
**Study design.** In the antioxidant study, asthmatic children were recruited from 1998 to 2000 and followed during 12 weeks with spirometric tests during the follow-up. At baseline, children were randomly assigned to either a placebo or a supplementation group (vitamin C 250 mg/day and vitamin E 50 mg/day). In the EVA study, asthmatic children were recruited and followed during 16 weeks from 2003 to 2004. Every two weeks children had a spirometric test. In both studies, children provided blood sample at baseline.

The study methods have been described previously [10]. In brief, 158 asthmatic children were recruited through the Hospital Infantil “Federico Gómez” (México City, México) between October 1998 and April 2000. At baseline, children randomly received either supplement (vitamin C 250 mg + vitamin E 50 mg per day) or placebo in a double-blind manner over a 12-week period follow-up. We also collected blood samples and diet information at baseline. We henceforth refer to this study as “Antioxidants”. In the “EVA”^b^ study, 158 asthmatic children were recruited from June 2003 to June 2005 at the same hospital under a similar protocol [11], but with no antioxidant intervention. After combining the two studies, a total of 257 participants with an adequate DNA sample and diet information were available for analysis. In the “Antioxidants” study, children performed two spirometric tests per week during 12 weeks. Children who participated in the “EVA” study performed spirometric tests every two weeks during 16 weeks. Spirometry was performed in accordance with the American Thoracic Society (ATS) specification [12] using an Easy One spirometer (NDD Medical Technologies, Andover, MA, USA). Children underwent skin testing to determine atopy. Given that the maximum site of effect of acute exposure to ozone in the human lung is at the level of small airways [13], and since a variety of epidemiological studies have reported an association between ozone exposure and impaired function of the small airways [10,14,15], the outcome of interest was FEF_25-75_. Over the follow-up we collected 4,548 measurements of FEF_25-75_ (Figure 1). Parents provided written informed consent. The studies protocols were reviewed and approved by ethics committees at the Instituto Nacional de Salud Publica, Hospital Infantil de México, and the National Institute of Environmental Health Sciences.

Asthma status and severity were confirmed by a pediatric allergist using Global Initiative for Asthma (GINA) guidelines [16]. Severity was rated as intermittent or persistent (mild, moderate, or severe). Ozone exposure (1-h maximum) and daily temperature were characterized from the measurements at the monitoring station closest to each child’s home.

### Diet

In the “Antioxidants” cohort, at baseline, children were randomly assigned (double-blind) to receive either placebo or supplement (vitamins C and E). EVA was an observational study; thus, there was no controlled supplementation.

Individual dietary intake was assessed using a version of the validated food frequency questionnaire developed by Willett [17] adapted to the Mexican population. Vitamin C intake was adjusted for total caloric intake [18]. Intake was increased by 250 mg/day for children assigned to the supplementation group or by the reported intake for those taking supplements.

### Genotyping

Genotyping was performed for the *GSTM1*, *GSTP1* (rs1695), and *NQO1* (rs1800566) polymorphisms, using polymerase chain reaction-based methods. 257 children were genotyped for *GSTM1* and *GSTP1*; a subset of 199 children was also genotyped for *NQO1*, having participated in a parallel study of nuclear families [19]. In 160 of the asthmatic children with additional genotype data available, individual ancestry proportions were estimated using the program FRAPPE [20] based on three ancestral populations: Native-American, European, and African [21].

#### Statistical analysis

Since children from the EVA cohort did not receive antioxidant supplementation, they were considered similar to children taking placebo in the Antioxidant cohort. We verified that these two groups did not differ in genotype frequencies. Vitamin C intake was dichotomized in this combined group using the median intake (105 mg/d). Children receiving supplementation, as part of the “Antioxidant” study, were classified into a higher intake group which had more than 280 mg/d. Thus, according to the distribution of vitamin C intake, we had three levels: (30–105), (>105-226), and (280-477 mg/day), hereafter referred to as “low”, “medium”, and “high” vitamin C, respectively.

We first analyzed the relationship between ozone exposure and FEF_25-75_ separately for each genotype of the three genes separately. Given that the relationships between the outcome and the concentrations of ozone in children with 1 or 2 *GSTM1* copies were similar each other but different to the observed in children with 0 copies (null genotype), we collapsed GSTM1 into null and positive. A similar situation was observed for GSTP1 and NQO1 genotypes. Thus, we defined “at genetic risk” (GR) and “not at genetic risk” (NGR) groups as follows: *GSTM1* null versus positive; *GSTP1* Val/Val versus Ile/Val + Ile/Ile, and *NQO1* Pro/Pro versus Pro/Ser + Ser/Ser.

After combining genotype and vitamin C intake, six strata were formed: (GR-low diet), (GR-medium), (GR-high), (NGR-low), (NGR-medium), and, (NGR-high) for each gene.

Subsequently, genes were grouped to create a putative antioxidant susceptibility genetic risk score by counting the number of risk alleles. Genotype score analysis included only children with complete genotype for all genotypes (n = 199). The creation of this index is detailed in Additional file 1: Table S1. We first explored the relationship between the outcome and ozone exposure within four genetic risk score groups collapsed because of sample size: 1 or 2 risk alleles; 3; 4; and 5 or 6. Since we observed that children with 3 risk alleles had similar response to the 1–2 risk alleles group and children with 4 risk alleles had similar response to children in the 5–6 risk allele group, we formed only two groups: 1 to 3, and 4 to 6 risk alleles. Additionally, given that no significant effect of ozone on FEF_25-75_ was observed in children with “medium” or “high” vitamin C groups, they were combined in a single “high” level for greater stability of estimates.

Thus, from the combination between the two susceptibility categories (1 to 3 or 4 to 6 risk alleles) and the two levels of vitamin C intake (low-high), four strata were formed: (1 to 3-low), (1 to 3-high), (4 to 6-low), and (4 to 6-high).

We analyzed all asthmatic children together and also grouped children with mild-persistent, moderate-persistent, and severe-persistent asthma into a “persistent” asthmatic group, which was analyzed separately.

To investigate whether vitamin C intake plus genotype modulates the relationship between ozone exposure and lung function, stratified linear mixed models with random intercept and random slope on ozone were fit in the whole sample and into the “persistent” group alone as well.

The stratified models were adjusted for age, gender, body mass index, height, time, cohort (Antioxidants or EVA), use of bronchodilator, and previous day minimum temperature. Previous day’s 1-h maximum ozone was the exposure. Effect estimates were calculated per interquartile range (IQR), which was 60 ppb. Genotype effect was calculated as the difference in coefficients for FEF_25-75_ in relation to ozone by genotype group. Diet effect was calculated as the difference between these coefficients between vitamin C groups.

Potential confounding by admixture was examined using exclusively the subsample of 160 children with ethnicity information. The association between each polymorphism and FEF_25-75_ was assessed through two linear mixed models. The first model was adjusted for age, gender, body mass index, height, time, cohort, use of bronchodilator, and previous day minimum temperature. The second model was also adjusted for Native-American ancestry but ancestry was not associated with the outcome and thus was not a confounder and was not retained in final models. Analysis was conducted using R software (version 2.9.1; the R foundation for Statistical Computing).

## Results

The final sample was composed of 144 (56%) participants from “Antioxidants” and 113 (44%) from “EVA” study. Major baseline characteristics of the study population are shown in Table 1. One third of the sample (30.4%) had antioxidant supplementation in the randomized controlled trial [10]. Persistent asthma was diagnosed in 164 of 257 children (63.8%). The prevalence of the *GSTM1* null genotype was 36.2%; 33.1% of children had the *GSTP1* Val/Val; and 33.7% had the *NQO1* Pro/Pro. Baseline characteristics, as well as ozone exposure and vitamin C intake, were homogeneously distributed among the genotypes of each polymorphism (Additional file 1: Table S2). Average ancestry proportions were 70.9% Native-American, 26.2% European, and 2.9% African.

**Table 1:** Characteristics of the study population of 257 asthmatic children residing in Mexico City 1998-2004

**Characteristic**	% or median(IQR)*
Gender (%male)	63.4
Age (years)	9.0 (7.2-11.0)
Height (cm)	134 (123–145)
BMI	18.5(16.1-21.6)
Antioxidant Study participants (%)	56.0
**Vitamin C intake**	
Supplementation group (%)	30.4
Vitamin C intake in supplementation group (mg/d)	346 (329–375)
Vitamin C intake out of supplementation group (mg/d)	105 (84–131)
**Asthma severity**	
Moderate- and severe-persistent asthma (%)	39.3
Mild-persistent asthma (%)	24.5
Mild-intermittent asthma (%)	36.2
Atopy (%)Ϫ	83.7
**Genotype and allele frequency**	**n**(%)
GSTM1 (Number of copies) †	
0	93 (36.2)
1	129 (50.2)
2	35 (13.6)
**GSTP1 rs1695**†	
Val/Val	85 (33.1)
Ile/Val	113 (44.0)
Ile/Ile	59 (22.9)
Ile/Ile + Ile/Val	172 (66.9)
NQO1 rs1800566 ‡	
Pro/Pro	67 (33.7)
Pro/Ser	88 (44.2)
Ser/Ser	44 (22.1)
Pro/Ser + Ser/Ser	132 (66.3)
**Ethnicity** (%) §	Mean (SD)
Native-American	70.9 (13.3)
European	26.2 (12.4)
African	2.9 (2.1)

†n = 257; ‡n = 199; § n = 160.

*Median(Q_25_-Q_75_); Ϫ Defined by skin test.

### Ozone exposure

The 1-h maximum ranged from 10 to 309 ppb with a mean of 96.9 ppb. The Mexican standard (110 ppb 1 h maximum) was exceeded on 30.7% of the days. (Additional file 1: Table S3).

#### Ozone effects by genotype group –All asthmatics

We found no overall effect of ozone on FEF_25-75_ (−3.3 ml/s; p = 0.76). In analyses by genotype, asthmatic children with the *GSTM1* null genotype appeared to experience a stronger ozone effect on lung function (−25.8 ml/s; p = 0.13; n = 93) than children with one (3.9 ml/s; p = 0.80; n = 129) or two (22.2 ml/s: p = 0.48; n = 35) copies. These results suggest a positive linear trend between the number of *GSTM1* copies and the average change in FEF_25-75_ in response to ozone exposure (p-trend = 0.09). We did not identify a potential susceptible group by *GSTP1* genotypes. (Additional file 1: Table S4).

#### Ozone effects by genotype group -persistent asthmatics

We found no overall effect of ozone on FEF_25-75_ (−16.2 ml/s; p = 0.55). Children with no *GSTM1* copy appeared to have stronger ozone-induced FEF_25-75_ decrements (−36.6 ml/s; p = 0.05; n = 65) than children with one (−15.0 ml/s; p = 0.42; n = 81) or two copies (23.7 ml/s; p = 0.59; n = 18) (p-trend = 0.10). We did not identify a potential susceptible group by *GSTP1 or NQO1* genotypes (Additional file 1: Table S4).

### Vitamin C intake

Recommended Daily Intake (RDI, 45 mg/day) by gender and age was exceeded for all but one child.

#### Ozone effects by Vitamin C -All asthmatics

No significant effect of ozone was observed through the different levels of vitamin C intake.

#### Ozone effects by vitamin C -persistent asthmatics

Children in the lowest vitamin C intake group (≤ 105 mg/day) and with “persistent” asthma had average decrement in FEF_25-75_ per 60 ppb ozone of −61.2 ml/s (p = 0.02). No significant effect of ozone was observed with higher vitamin C intake (Additional file 1: Table S5).

### Effect of ozone on FEF_25-75_ stratified by genotype for each of the three antioxidant genes and vitamin C intake

#### Ozone effects by genotype and vitamin C -All asthmatics

In the whole sample, children with low vitamin C intake and with *GSTM1* null genotype had the greatest average decrement in FEF_25-75_ (−69.0 ml/s; p = 0.07). Smaller effects of ozone on FEF_25-75_ were observed in other *GSTM1*-diet strata and in other *GSTP1*- or *NQO1*-diet combinations. The genotype effect of GSTM1 decreased as the level of vitamin C increased (p-trend < 0.01) (Table 2).

**Table 2:** **Effect of ozone on FEF**_**25-75**_, **according to genotype and vitamin C intake **^&^

	***Vitamin C intake***					
	**30–105 mg/day**		**>105–226 mg/day**		**280**-**477 mg/day**	
**Genotype**	**n**	**Coeff(95% CI)**	**n**	**Coeff(95% CI)**	**n**	**Coeff(95% CI)**
GSTM1						
*All asthmatics*						
Null	28	−69.0 (−144.0, 6.0)£	34	−16.2 (−66.8, 34.4)	31	−13.8 (−58.5, 30.9)
Positive	57	−12.6 (−57.3, 32.1)	60	22.2 (−23.7, 68.1)	47	6.9 (−38.9, 52.8)
Genotype effect ^Ω^		56.4 (−138.0, 27.0)		38.4 (−31.2,108.0)		20.7 (−43.2, 84.6) *
*Persistent asthmatics*						
Null	17	−91.2 (−184.1, 1.7)£	24	−23.4 (−81.0, 34.2)	24	−13.2 (−77.9, 51.5)
Positive	28	−35.4 (−102.4, 31.6)	36	−15.6 (−69.7, 38.4)	35	13.8 (−40.3, 67.9) **
Genotype effect		55.8 (56.4, -168.0)		7.8 (−114.0, 84.0)		27.0 (−57.6, 111.6)
GSTP1 rs1695						
*All asthmatics*						
Val/Val	27	−43.2 (−106.7, 20.3)	31	2.4 (−56.4, 61.2)	27	3.6 (−65.8, 73.0)
Ile/Val + Ile/Ile	58	−24.0 (−74.6, 26.6)	63	7.8 (−32.2, 47.8)	51	3.6 (−35.2, 42.4)
Genotype effect		19.2 (−68.4, 106.8)		5.4 (−65.4, 76.2)		0.0 (−79.8, 79.8)
*Persistent asthmatics*						
Val/Val	13	−82.8 (−167.5, 1.9)£	21	−33.0 (−104.7, 38.7)	20	18.2 (−67.8, 103.8) *
Ile/Val + Ile/Ile	32	−54.0 (−119.9, 11.9)	39	−12.0 (−59.0, 35.0)	39	0.0 (−49.4, 49.4)
Genotype effect		28.8 (−96.6, 154.2)		21.0 (−64.8, 106.8)		−18.2 (−117.0, 81.0)
NQO1 rs1800566*						
*All asthmatics*						
Pro/Pro	24	−55.2 (−134.0, 23.6)	22	50.4 (−17.8, 118.6)	21	−37.8 (−114.2, 38.6)
Pro/Ser + Ser/Ser	36	−4.8 (−62.4, 52.8)	42	−10.8 (−56.7, 35.1)	54	10.8 (−29.2, 50.8)
Genotype effect		50.4 (−45.4, -144.0)		−61.2(−141.6, 192.0)		48.6 (−31.2,126.0)
*Persistent asthmatics*						
Pro/Pro	14	−75.0 (−182.0, 32.0)	13	−0.3 (−69.7, 69.1)	19	−39.6 (−119.6, 40.4)
Pro/Ser + Ser/Ser	23	−42.0 (−105.5, 21.5)	31	−17.4 (−70.3, 35.5)	37	17.4 (−32.0, 69.0) **
Genotype effect		33.0 (−84.0,150.0)		−17.1(−111.6, 77.4)		57.0(−32.8,146.4)

Models were adjusted for gender, age, BMI, height, time, cohort, use of bronchodilator, and minimum temperature. All asthmatics: n = 257 and 4548 repeated measures; persistent (mild, moderate, or severe) asthmatics: n = 164 and 3029 repeated measures.

*NQO1 models: all asthmatics n = 199 and 3842 repeated measures; persistent (mild, moderate, or severe): n = 137 and 2715 repeated measures. € p ≤ 0.05; £ 0.05 < p < 0.1; Ω Genotype effect is defined to be the change in response produced by a change in the level of genotype.

*p-trend ≤ 0.05; ** 0.05 < p-trend < 0.1.

^&^FEF_25-75_ is reported as ml/s per 1-h 60 ppb on the day prior to spirometric test.

#### Ozone effects by genotype and vitamin C -persistent asthmatics

**Figure 2:**
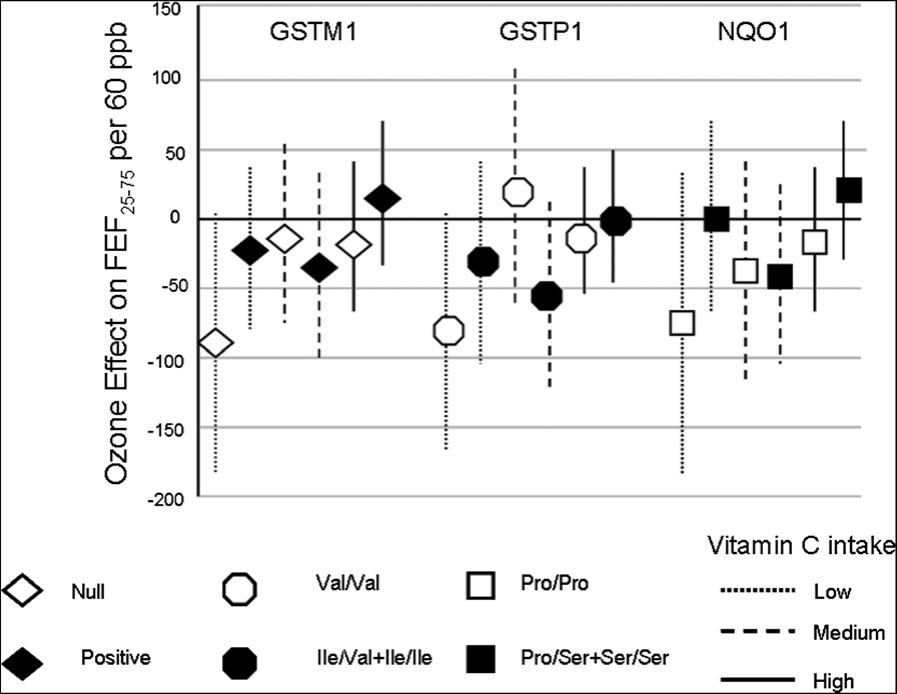
**Effect of ozone on FEF**_**25**-**75**_** and 95% CI according to genotypes and vitamin C intake.** Persistently asthmatic children with vitamin C intake between 30 to 105 mg/day and with *GSTM1* null had lower levels of FEF_25-75_ than children with either *GSTM1* positive genotype or dietary vitamin C intake higher than 105 mg/day. Similar situation was observed for Val/Val vs. Ile/Val + Ile/Ile GSTP1 polymorphisms. Linear mixed models were adjusted for age, gender, BMI, height, cohort, time, use of bronchodilator, and previous day’s minimum temperature. Reported values correspond to the effect on FEF_25-75_ per 60 ppb of ozone.

When we restricted the analysis to the “persistent” asthma group, the effect of ozone was more pronounced in the stratum with low vitamin C intake. The average decrement in FEF_25-75_ was −91.2 ml/s (p = 0.06) (Figure 2). Children with the Val/Val *GSTP1* genotype and with low vitamin C intake had an average decrement in FEF_25-75_ of −82.8 ml/s (p = 0.06) per 60 ppb of ozone. We did not find notable ozone effects on FEF_25-75_ in the different strata of diet-*NQO1* genotypes (Table 2).

### Effect of ozone on FEF_25-75_ by genotype score and vitamin C intake

Only 7 children (3.5%) had all 6 risk alleles (no *GSTM1* copies, Val/Val, and Pro/Pro genotypes) and 48.3% had 4 or more risk alleles (Additional file 1: Table S1).

#### Ozone effects by genotype score and vitamin C -All asthmatics

We found that among all asthmatics, the effect of ozone on FEF_25-75_ in children with 4 to 6 risk alleles and low vitamin C intake was stronger (−58.8 ml/s; p = 0.07) than in those with other gene-diet combinations (Table 3).

**Table 3:** **Effect of ozone on FEF**_**25-75**_, **according to the number of risk alleles and vitamin C **^&^

	**30–105 mg/day**		**>105–477 mg/day**		
**Risk Alleles**	**n**	**Coeff(95% CI)**	**n**	**Coeff(95% CI)**	**Diet effect**^**π**^
*All asthmatics*					
1 to 3	30	24.0 (−50.0, 98.1)	73	27.0 (−16.5, 70.5)	3.0 (−82.2, 88.2)
4 to 6	30	−58.8 (−122.3, 0.1) £	66	−16.8 (−49.7, 16.1)	42.0(−24.0, 108.0)
Genotype effect ^Ω^		−82.8 (−180.0, 15.0) £		−43.8 (−98.4, 11.4)	
*Persistent asthmatics*					
1 to 3	18	19.2 (−67.8, 106.2)	50	15.6 (−35.0, 66.2)	−3.6 (−105.6, 98.4)
4 to 6	19	−**97**.**2** (−**185**.**4**, -**9**.**0**) €	50	−18.6 (−58.6, 21.4)	78.6 (77.1, 80.1) £
Genotype effect		−116.4 (−118.4, -114.4) £		−34.2 (−98.4, 30.0)	

Models were adjusted for gender, age, BMI, height, chronological time, cohort, use of bronchodilator, and minimum temperature. All asthmatics: n = 199 and 3842 repeated measures; persistent (mild, moderate or severe) asthmatics: n = 137 and 2715 repeated measures.

€ p ≤ 0.05; £ 0.05 < p ≤ 0.1; Ω Genotype effect is defined to be the change in response produced by a change in the level of genotype.

Π Diet effect is defined to be the change in response produced by a change in the level of diet intake. ^&^FEF_25-75_ is reported as ml/s per 1-h 60 ppb on the day prior to spirometric test.

#### Ozone effects by genotype score and vitamin C -persistent asthmatics

**Figure 3:**
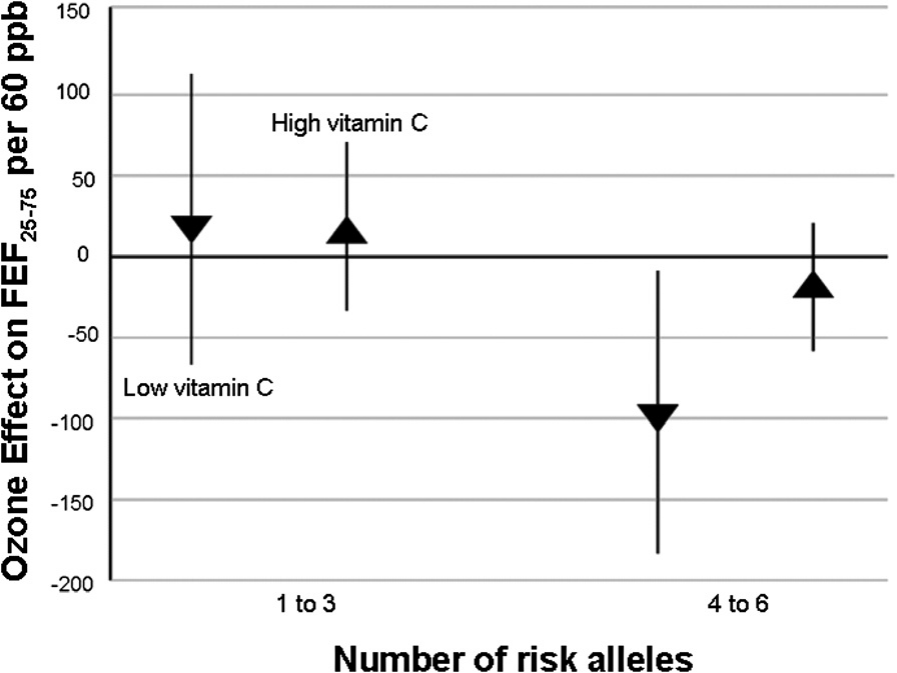
**Effect of ozone on FEF**_**25**-**75**_** and 95% CI according to genotype score and vitamin C intake.** Persistently asthmatic children with vitamin C intake between 30 to 105 mg/day (low diet) and with 4 to 6 risk alleles had lower levels of FEF_25-75_ than children with either *1* to 3 risk alleles or dietary vitamin C intake higher than 105 mg/day (high diet). Linear mixed models were adjusted for age, gender, BMI, height, cohort, time, use of bronchodilator, and previous day’s minimum temperature. N = 137 asthmatic children and 2715 repeated measures.

When we restricted the analysis to the “persistent” asthma group, the effect of ozone in children with 4 to 6 risk alleles and low vitamin C was even more pronounced (−97.2 ml/s; p = 0.03) than in the whole sample and stronger than the effects on either children with 3 to 6 risk alleles or with high level of vitamin C intake (Figure 3).

Looking at the low vitamin C intake group, the difference between the average decrements of children with 1 to 3 risk alleles against children with 4 to 6 was −116.4 ml/s (p = 0.08). Looking at the group with 4 to 6 risk alleles, the difference between the average decrements of children with low against high vitamin C intake, was 78.6 ml/s (p = 0.07) Table 3.

## Discussion

We found suggestive evidence that the acute effects of ozone exposure on asthmatic children’s pulmonary function may depend on both antioxidant genes and dietary intake of vitamin C. These ozone effects appear more pronounced in persistent asthmatic children. In addition, the cumulative effect of genetic susceptibility in *GSTM1*, *GSTP1*, and *NQO1* polymorphisms and low intake of vitamin C was associated with the largest decrement in FEF_25-75_ among children with persistent asthma.

*GSTM1* has been most intensively studied as a deleted versus a nondeleted dichotomous polymorphism; there are few data using *GSTM1* copy number. In this study, we found a potential dose–response effect of *GSTM1* on the relationship between FEF_25-75_ and ozone exposure. This result is consistent with the dose–response relationship between *GSTM1* and the risk of lung cancer on smokers [22].

In human lung cells, *GSTP1* Val/Val genotype has been associated with reduced GST Pi enzymatic activity [23]. Our finding that the Val/Val may confer higher susceptibility to ozone damage than Ile/Val or Ile/Ile genotypes is consistent with that result. In addition, some epidemiologic studies have reported associations between Val/Val genotype and reduced lung function in young adults [24] and increased risk of early onset of asthma [25,26]. Discrepancies with other studies [27,28] that report the Val allele as protective may be related to ethnic differences among populations, with their consequent differences in linkage disequilibrium patterns, or due to change variation among studies.

Regarding diet, persistent asthmatics with low daily vitamin C intake had significant decrements in FEF_25–75_ associated to ozone exposure, but the effect of ozone was lessened among children with vitamin C intake over 105 mg/day. Our results suggest that persistent asthmatic children exposed to ozone might benefit from vitamin C intake above the RDI. Intake of > 105 mg/day was reached in our study from diet alone. This suggests that children do not need to take supplements. Consumption of daily fruits and vegetables may provide enough vitamin C for lessening the effects of ozone, on lung function, regardless the combination of antioxidant genotypes. Furthermore, fruits and vegetables provide additional nutrients that supplements do not include.

When genetic and dietary data were combined, persistent asthmatics with both low vitamin C intake and *GSTM1* null or Val/Val *GSTP1* had, on average, lower levels of FEF_25–75_ than other children.

Although our selection of 105 mg/day as the cutoff was based on the data distribution, it is according with the increment of 100 mg/d suggested by some studies for increasing about 10-50 ml in FEV_1_[29].

Using our genotype score as an index of genetic susceptibility, we found that the effect of ozone on persistent asthmatics with less than 105 mg/day vitamin C and with 4 to 6 risk alleles was slightly stronger than that estimated for *GSTM1* under the single gene approach in the same dietary group (−97.2 ml/s against −91.2 ml/s respectively). The difference between both effects was not significant (p = 0.88). This result suggests that the *GSTM1* genotype alone could have been used as an indicator of susceptibility to ozone.

As a result of environmental health policies in Mexico City, concentrations of ozone have been reduced over time. From 1998 to 2000 (first cohort enrollment), the average concentrations were between 100 and 125 ppb; while by the second cohort enrollment (2003–2005), concentrations were between 85 to 100 ppb [30]. When we fit separated models by cohort, we found that in the first cohort, the effect of ozone on children with low vitamin C intake and *GSTM1* null genotype, was −96.0; 95% CI(−204.0,12.6); whereas a lower effect was observed in the second cohort (−20.4; 95% CI (−114.6, 73.2)). Thus, final models were adjusted for a covariate accounting for the cohort.

Although our focus was on FEF_25-75_, we also analyzed the association between ozone and other measures of lung function including FEV_1_, FVC and PEF but we did not find any significant effect.

Regarding to the assessment of the potential confounding role of ethnicity, we rejected the null hypothesis of association between Native-American ancestry and the outcome setting an alpha-level of 0.20 instead of 0.05 level in order to insure adequate power to detect any important confounder effect [31]. This lack of confounder effect may be explained by the fact that the children were recruited at the same Public Hospital suggesting similar socioeconomic status.

Some advantages and limitations of our study need to be addressed. Advantages include repeated measurements of FEF_25-75_. Obtaining longitudinal measurements of quantitative phenotypes reduces potential outcome misclassification and increases power by focusing on within-subject variations in outcome while controlling for among subjects differences using random intercepts and random slopes on time-dependent ozone exposure.

We were able to assess the potential confounding effect of population admixture and thereby exclude any potential confounding effect of admixture from our data. This is the first time that the confounding role of ethnicity in the association between genotype and respiratory health of Mexican asthmatic children exposed to ozone has been assessed. Ethnicity was based on genome-wide association genotyping. The absence of association between the levels of FEF_25-75_ and ethnicity in these Mexican asthmatic children supports the validity of previously-reported genetic associations in this population [9,32].

Potential limitations include inaccuracy in assessment of personal ozone exposure. Because exposure assessment was based on monitoring stations located within 5 km from the children’s homes, potential misclassification of exposure is possible. However, using personal monitors on a subset of participants in the “Antioxidants” study (n = 144), a significant association (p < 0.01) between personal and ambient ozone levels was observed [33]. Based on this validation, we would expect any misclassification in exposure to be random with the consequence of underestimating the adverse effect of ozone.

We were not able to measure plasma vitamin C levels of the participants but a validated food frequency questionnaire was used to estimate intake. Self-reported vitamin C intake was adjusted for total caloric intake. Through this adjustment, the between-person variation due to over- or under reporting of intake is reduced and a gain in accuracy is potentially obtained [18,34]. Under this assumption, we would expect any misclassification in vitamin C intake to be random; therefore its potential protective effect may be underestimated.

While we focused on dietary vitamin C intake, we recognize that vitamin E has also been positively related to lung function. Children in the highest level of vitamin C intake had 50 mg per day of vitamin E as part of the antioxidant supplementation. It is worthy to note that although children in the medium level of vitamin C did not have supplementation, they had no significant ozone effects. Therefore, the effect of vitamin C might be predominant. Nevertheless, since both vitamin C and E may be present in the same foods, and because the biological interplay between them [35], part of the effect observed for vitamin C may be related to vitamin E intake as well.

Although ozone has received a great deal of attention, PM_2.5_ has also been associated with respiratory diseases. However, since concentrations of PM_2.5_ in México City only began to be registered by the network in 2000, we were not able to analyze the effect of this pollutant. Nevertheless, when data were available, it has been observed that PM_2.5_ and ozone are correlated (r = 0.46; p < 0.0001) [11]. Thus, part of the effect observed for ozone may be related to PM_2.5_ exposure.

Selection bias is unlikely because those children without diet or genotype information were not aware of their individual susceptibility to ozone exposure. In addition, included and excluded groups had similar characteristics. Moreover, we used an objective measure of ozone effect – pulmonary function.

We acknowledge that our sample size was relatively small. Large sample sizes are required to study multi-way interactions with complex diseases such as asthma. In contrast, longitudinal studies of quantitative traits with repeated measurements are more powerful than cross-sectional studies of binary disease outcomes. Thus, despite our modest sample size, we were able to examine effects of ozone and diet on repeated measures analyses of the quantitative traits of FEF_25-75_ in our asthmatic children.

Although we are not aware of a study with similar design that could be used for replication, a number of observational studies suggest that vitamin C may reduce asthma risk and may prevent inflammatory response in the airways by reducing reactive oxygen species [36]. Further, the intake of vitamin C and of fruits rich in vitamin C has been positively associated to lung function [35]. Moreover, the protective effect of high fruits and vegetables intake has been reported as having potentially inverse modifying influence to cigarette smoking on the asthma risk for genetically vulnerable individuals [37] and the antioxidant role of some genes such as *GSTM1*, *GSTP1*, and *NQO1* has been recognized [6,7,26].

Albeit reported p-values throughout this paper have not been corrected for multiple comparisons, we believe that it is unlikely to detect effects due to statistical fluctuations only because of the epidemiological evidence for the interactive effects of air pollutants with vitamin C intake [29,35,38] and antioxidant genes [25,27,28] on asthma symptoms and lung function.

## Conclusions

We conclude that dietary vitamin C intake, either from fruits and vegetables or supplementation provides some protection against the effect of ozone on the pulmonary function of asthmatic children in an area with high ozone exposures– Mexico City. Effects of ozone were predominantly seen in the lowest tertile of vitamin C intake – up to 105 mg/day. Daily consumption of a slice of papaya or pineapple and 2 oranges, for example, can provide more than 105 mg/day of vitamin C. Dietary modification to increase intake of fruits rich in vitamin C is a simple intervention that is likely to have other health benefits. Ozone may have a greater impact on the respiratory health of children with deficient enzymatic activity on antioxidant genes and with low vitamin C intake than on that of children who have adequate enzymatic activity, vitamin C intake, or both.

## Endnotes

^a^ Normal metabolic processes in all cells are the main endogenous sources of reactive oxygen species.

^b^ “Emission vehicular and asthma” in Spanish.

## Abbreviations

GSTM1: Glutathione S-tranferase M1; GSTP1: Glutathione S-tranferase P1; NQO1: Nicotinamide adenine dinucleotide (phosphate) reduced: Quinone Oxidoreductase; FEF_25-75_: Forced Expiratory Flow between 25 and 75 s; EVA: Emission vehicular and asthma.

## Competing interests

The authors declare that they have no competing interests.

## Authors’ contributions

HM, Conducted the data analysis, interpretation of the results and writing of the manuscript; DD, directed the data analysis and the writing of the paper JS, Directed the data analysis; DG, Directed the data analysis and writing of the paper; NL, directed the data analysis and writing of the paper; JJS, Participated in the standardization of lung testing and data collection; BD, Participated in the standardization of lung testing and data collection MR, participated in the protocol, data collection, standardization, and realization of lung testing; AB, participated in the protocol, data collection, standardization, and realization of lung testing; HL, Conducted the genotyping process; SJL, Developed the protocol, obtained funding for the project, directed the genotyping process, and directed the writing of the manuscript IR, Developed the protocol, obtained funding for the project, and directed the data analysis and the writing of the paper. All authors read and approved the final manuscript.

## Supplementary Material

Additional file 1: Table S1 Creation of genotype score by counting the number of risk alleles. **Table S2.** Basal characteristics of the study population. **Table S3.** Air pollution levels during the study from the Mexico City monitoring network, 1998-2004. **Table S4.** Effect of ozone on FEF_25–75_ (per 1-hr 60 ppb on the day prior to spirometric test) according to genotype. **Table S5.** Effect of ozone on FEF_25–75_ (per 1-hr 60 ppb on the day prior to spirometric test) according to vitamin C intake. Click here for file
