# Supplementary material for: Ozone exposure, vitamin C intake, and genetic susceptibility of asthmatic children in Mexico City: a cohort study
Source: Respir Res. 2013 Feb 4;14(1):14. doi: 10.1186/1465-9921-14-14 (PMC3579760; doi:10.1186/1465-9921-14-14)
Supplement: Additional file 1: Table S1 — Creation of genotype score by counting the number of risk alleles. Table S2. Basal characteristics of the study population. Table S3. Air pollution levels during the study from the Mexico City monitoring network, 1998-2004. Table S4. Effect of ozone on FEF25–75 (per 1-hr 60 ppb on the day prior to spirometric test) according to genotype. Table S5. Effect of ozone on FEF25–75 (per 1-hr 60 ppb on the day prior to spirometric test) according to vitamin C intake. [file 1465-9921-14-14-S1.docx]

**SUPPLEMENTARY MATERIAL**

**Material and Methods**

***Study population***

In Mexico City two cohort studies have been conducted with the aim to assess the effect of air pollution on the respiratory health of asthmatic children. In the “Antioxidants” study 158 children participated. Participants were recruited from 1998 to 2000 and followed during 12 weeks with two spirometric tests per week. At baseline and every 6 weeks children performed nasal lavages. At baseline, randomization to receive the supplement (250 mg/day of Vitamin C and 50 mg/day of Vitamin E) or placebo was conducted in a double-blind manner. On the other hand, in the “Emission vehicular and asthma” (EVA) study, 158 children participated. Children were recruited from 2003 to 2004 and followed during 16 weeks. Every two weeks children had a spirometric test and a nasal lavage. There was no intervention in this cohort. In both cohorts, children residents of Mexico City were recruited through the allergy clinic of the Hospital Infantil “Federico Gomez”. For baseline information, in both studies, parents filled out a general-purpose questionnaire on sociodemographic, health and risk factors for asthma. In addition, parents filled out a validated food-frequency questionnaire regarding their child’s dietary and supplements intake. This questionnaire was adapted to the Mexican population from the questionnaire developed by Willett.

Asthma status and severity were confirmed by a pediatric allergist based on clinical symptoms and response to treatment according the Global Initiative for Asthma (GINA) guidelines. According to their severity status, children were classified as mild intermittent, mild persistent, moderate persistent or severe asthma.

***Spirometry***

The spirometric tests were performed according the American Thoracic Society (ATS) specifications (ATS 1995) (S1) using an Easy One spirometer (ndd Medical Technologies, Andover, MA, USA). The tests were conducted at around the same time in the morning in a room with stable temperature and relative humidity. All the lung functions were examined for quality by the same technicians, and the best of three technically acceptable maneuvers was selected for each test.

***Nasal lavage***

Nasal lavage was performed using the methodology proposed by Diaz-Sanchez and colleagues (S2). Subjects were seated with the nasopharynx closed while leaning the neck back 45° from the vertical. 5 ml of pre-warmed (37° C) normal saline solution was delivered by a pipette into each nostril, and then the subjects softly shook their head from side to side for 10 seconds. Bringing the head forward, the subject expelled the wash fluid into a plastic receptacle. The maneuver was repeated up to four additional times at 30-sec intervals, with each wash being collected into a separate tube. IL-8 levels were obtained from the nasal lavage using enzyme-linked immunosorbent assay ELISA kits. Total cell counts were determined through a hematocymeter and differentials were determined by prepared cytocentrifuge slides using 2.5 ml. of the unprocessed nasal lavage fluid.

***Genotyping***

DNA was extracted from the children and the parents using a standard GentraPuregene protocol (Gentra Systems Minneapolis, MN, USA) from whole blood. The *GSTM1* gene was detected by real time Polymerase Chain Reaction (PCR) that distinguishes among 0, 1, or 2 allele copies. The three genotypes for the single nucleotide polymorphisms (SNP) rs1695 on *GSTP1* genotypes (Ile/Ile, Ile/Val or Val/Val) were determined by a taqman allelic discrimination method. Standard PCR cycling conditions were used. Allele-specific PCR products were detected on an ABI 7700 (Applied Biosystems) and clustered by genotype using Sequence Detection System software (Applied Biosystems). Ambiguous samples were manually clustered and verified by sequencing. *NQO1* (rs1800566) genotypes were determined by PCR fragment length polymorphism. As quality control, assays were repeated on 5% of the samples, and the replicates were 100% concordant.

***Statistical Methods***

Socio-demographic characteristics, potential indoor environmental exposures, dietary vitamin C intake, respiratory outcomes and admixture were compared according *GSTM1, GSTP1* and *NQO1* genotypes by bivariate analyses using either Mann-Whitney or the chi-square test for continuous or categorical variables respectively.

The ozone effect over the different gene-diet strata was estimated using linear mixed models for quantitative outcomes with both random intercept and random slope on ozone (time dependent variable). Models for FEF_25-75_ were adjusted for potential confounding factors including age, gender, height, body mass index (BMI), previous day minimum temperature, time, use of bronchodilator, and cohort. The nonlinear relation between FEF_25-75_ and height was captured through a smooth function.

Other variables such as maternal schooling, exposure to environmental tobacco, pets at home and bronchodilator use were not significant.

**References**

S1.Standardization of Spirometry, 1994 Update. American Thoracic Society. Am J Respir Crit Care Med 1995;152:1107-1136.

S2.Diaz-Sanchez D, Dotson AR, Takenaka H, Saxon A. Diesel exhaust particles induce local IgE production in vivo and alter the pattern of IgE messenger RNA isoforms. J Clin Invest 1994;94:1417-25.

Table S1. Creation of genotype score by counting the number of risk alleles

| GSTM1 | GSTP1 | NQO1 | COUNT(%) | #risk alleles | Cumulative  n (%) |
| --- | --- | --- | --- | --- | --- |
| 2copies | Ile/Ile | Ser/Ser | 0 | 0 |  |
| 2copies | Ile/Ile | **Pro**/Ser | 6 (3.0) | 1 |  |
| 2copies | Ile/**Val** | Ser/Ser | 3 (1.5) | 1 |  |
| **1copy** | Ile/Ile | Ser/Ser | 7 (3.5) | 1 | 16 (8.0) |
| 2copies | Ile/Ile | **Pro/Pro** | 3 (1.5) | 2 |  |
| 2copies | Ile/**Val** | **Pro**/Ser | 3 (1.5) | 2 |  |
| 2copies | **Val/Val** | Ser/Ser | 3 (1.5) | 2 |  |
| **1copy** | Ile/Ile | **Pro**/Ser | 8 (4.0) | 2 |  |
| **1copy** | Ile/**Val** | Ser/Ser | 6 (3.0) | 2 |  |
| **null** | Ile/Ile | Ser/Ser | 1 (0.5) | 2 | 24(12.0) |
| 2copies | Ile/**Val** | **Pro/Pro** | 2 (1.0) | 3 |  |
| 2copies | **Val/Val** | **Pro**/Ser | 5 (2.5) | 3 |  |
| **1copy** | Ile/Ile | **Pro/Pro** | 9 (4.5) | 3 |  |
| **1copy** | Ile/**Val** | **Pro**/Ser | 28 (14.1) | 3 |  |
| **1copy** | **Val/Val** | Ser/Ser | 3 (1.5) | 3 |  |
| **null** | Ile/Ile | **Pro**/Ser | 6 (3.0) | 3 |  |
| **null** | Ile/**Val** | Ser/Ser | 10 (5.0) | 3 | 63(31.7) |
| 2copies | **Val/Val** | **Pro/Pro** | 4(2.0) | 4 |  |
| **1copy** | Ile/**Val** | **Pro/Pro** | 17(8.5) | 4 |  |
| **1copy** | **Val/Val** | **Pro**/Ser | 11 (5.5) | 4 |  |
| **null** | Ile/Ile | **Pro/Pro** | 4 (2.0) | 4 |  |
| **null** | Ile/**Val** | **Pro**/Ser | 13 (6.5) | 4 |  |
| **null** | **Val/Val** | Ser/Ser | 11 (5.5) | 4 | 60(30.2) |
| **1copy** | **Val/Val** | **Pro/Pro** | 11 (5.5) | 5 |  |
| **null** | Ile/**Val** | **Pro/Pro** | 10 (5.0) | 5 |  |
| **null** | **Val/Val** | **Pro**/Ser | 8 (4.0) | 5 | 29(14.6) |
| **null** | **Val/Val** | **Pro/Pro** | 7 (3.5) | 6 | 7(3.5) |

Risk alleles are in bold

**RESULTS**

Table S2. Basal characteristics of the study population

|  | GSTM1 (CNV)* | | |  | GSTP1* | | |  | NQO1† | | |  |
| --- | --- | --- | --- | --- | --- | --- | --- | --- | --- | --- | --- | --- |
| Genotype | 0 | 1 | 2 | p | Val/Val | Ile/Val | Val/Val | p | Pro/Pro | Pro/Ser | Ser/Ser | p-value |
| N (%) | 93 (36.2) | 129 (50.2) | 35 (13.6) |  | 85 (33.1) | 113 (44.0) | 59 (22.9) |  | 67 (33.7) | 88 (44.2) | 44 (22.1) |  |
| **ANTHROPOMETRY** | |  |  |  |  |  |  |  |  |  |  |  |
| Gender (%male)‡ | 66.7 | 61.2 | 62.9 | 0.71 | 60.0 | 67.3 | 61.0 | 0.52 | 55.2 | 67.1 | 72.7 | 0.13 |
| Age (years)§ | 9.0  [7.9-11.0] | 8.7  [7.0-11.0] | 9.2  [7.2-12.0] | 0.42 | 8.6  [7.0-11.0] | 9.0  [7.7-11.0] | 9.0  [7.0-11.0] | 0.83 | 8.0  [7.0-10.0] | 9.0  [7.0-11.0] | 9.0  [7.2-11.0] | 0.61 |
| Weight (kg)§ | 33.5  [27.8-43.0] | 33.0  (25.5-42.0) | 32  [25.0-45.0] | 0.98 | 31.5  [26.4-41.0] | 34.0  [27.0-44.0] | 34.0  [25.0-45.0] | 0.82 | 29.5  [26.0-38.0] | 33.8  [26.0-42.5] | 32.5  [27.0-43.0] | 0.13 |
| Height (m)§ | 1.36  [1.25-1.44] | 1.32  [1.22-1.45] | 1.33  [1.22-1.51] | 0.80 | 131  [122.0-145.0] | 135  [125.0-143.0] | 132  [123.5-148.0] | 0.92 | 1.30  [1.22-1.42] | 1.32  [1.23-1.45] | 1.35  [1.22-1.44] | 0.29 |
| BMI§ | 19.2  [16.3-22.1] | 18.4  [16.0-21.4] | 17.6  [15.6-20.4] | 0.43 | 18.1  [16.1-20.2] | 19.0  [16.1-22.2] | 18.6  [16-22.3] | 0.63 | 17.5  [15.4-19.8] | 18.5  [15.9-21.7] | 19.1  [16.3-21.2] | 0.25 |
| **Socio Economic Status (SES)** | |  |  |  |  |  |  |  |  |  |  |  |
| Maternal schooling (years) ll | 8.9 (2.7) | 9.5 (2.9) | 9.2 (4.0) | 0.38 | 9.0 (3.2) | 9.4 (3.0) | 9.4 (2.8) | 0.57 | 9.2 (3.4) | 9.4 (2.7) | 8.2 (2.9) | 0.10 |
| Paternal schooling (years) ll | 9.2 (2.9) | 9.9(3.6) | 9.6(3.2) | 0.38 | 9.7 (3.4) | 9.8 (3.3) | 9.1 (3.0) | 0.48 | 10.1 (3.6) | 9.1 (2.8) | 9.0 (3.3) | 0.11 |
| **ENVIRONMENTAL EXPOSURES** | |  |  |  |  |  |  |  |  |  |  |  |
| Maternal smoking at home(%)‡ | 26.9 | 32.0 | 28.0 | 0.76 | 27.9 | 26.8 | 38.1 | 0.40 | 17.2 | 26.1 | 29.0 | 0.36 |
| Paternal smoking at home(%)‡ | 54.3 | 55.2 | 55.2 | 0.99 | 55.3 | 56.5 | 51.1 | 0.83 | 46.7 | 50.0 | 66.7 | 0.13 |
| Pets at home (%)‡ | 59.1 | 55.7 | 45.7 | 0.40 | 54.1 | 53.2 | 62.5 | 0.49 | 55.2 | 57.0 | 60.5 | 0.86 |
| Humidity at home (%)‡ | 50.0 | 44.3 | 48.0 | 0.79 | 38.9 | 54.1 | 44.7 | 0.23 | 45.3 | 37.9 | 53.3 | 0.38 |
| Ozone (1 hr max ppb)§ | 96  [65-124] | 93  [63-121] | 96  [65-117] | 0.23 | 96  [65-124] | 95  [64-122] | 92  [62-118] | 0.19 | 96  [64-127] | 94  [62-120] | 94  [65-115] | 0.36 |
| **ANTIOXIDANT DIET** | |  |  |  |  |  |  |  |  |  |  |  |
| Supplementation group (%) | 33.3 | 27.9 | 31.4 | 0.68 | 31.8 | 32.7 | 23.7 | 0.45 | 31.3 | 42.1 | 38.6 | 0.39 |
| Vitamin C intake§ (mg/day)  (without controlled supplementation) | 107.7  [85.9-128.0] | 107.0  [84.8-132.1] | 104.3  [79.7-132.9] | 0.99 | 107.5  [84.8-120.8] | 107.7  [85.5-142.1] | 107.0  [81.2-135.7] | 0.99 | 103.8  [84.7-131.4] | 108.4  [78.9-135.3] | 105.5  [90.1-122..9] | 0.86 |
| Vitamin C intake§ (mg/day)  (supplementation group) | 346.4  [327.7-369.8] | 349.6  [327.6-375.9] | 349.3  [331.3-385.8] | 0.99 | 359.1  [344.6-400.3] | 337.0  [324.3-370.3] | 335.0  [322.6-364.8] | 0.41 | 338.8  [316.7-359.1] | 357.9  [333.3-385.8] | 341.7  [322.1-385.5] | 0.45 |
| **ASTHMA SEVERITY** | |  |  |  |  |  |  |  |  |  |  |  |
| Moderate persistent asthma (%)‡ | 47.3 | 34.9 | 34.3 |  | 36.5 | 38.1 | 45.8 |  | 46.3 | 46.6 | 43.2 |  |
| Mild persistent asthma (%)‡ | 22.6 | 27.9 | 17.1 |  | 27.1 | 25.7 | 18.6 |  | 22.4 | 22.7 | 25.0 |  |
| Mild intermittent asthma (%)‡ | 30.1 | 37.2 | 48.6 | 0.27 | 36.5 | 36.3 | 35.6 | 0.90 | 31.3 | 30.7 | 31.8 | 0.96 |
| Atopic | 68.1 | 65.6 | 71.4 | 0.79 | 67.1 | 64.0 | 74.1 | 0.41 | 57.6 | 61.4 | 69.8 | 0.44 |
| **ANCESTRY** |  |  |  |  |  |  |  |  |  |  |  |  |
| Native American | 70.7 (15.2) | 70.7 (12.0) | 71.7 (13.3) | 0.95 | 73.3 (10.9) | 71.4 (15.1) | 66.8 (11.5) | 0.08 | 68.7 (13.7) | 69.9 (11.7) | 75.3 (14.9) | 0.05 |
| European | 26.3 (14.1) | 26.4 (11.1) | 25.1 (12.1) | 0.91 | 24.0 (10.0) | 25.9 (13.9) | 29.6 (11.4) | 0.12 | 28.0 (12.8) | 27.2 (11.0) | 22.2 (13.6) | 0.06 |
| African | 2.9 (2.0) | 2.9 (1.9) | 3.2 (3.1) | 0.84 | 2.7 (1.5) | 2.8 (2.1) | 3.6 (2.6) | 0.07 | 3.3 (2.6) | 3.0 (1.9) | 2.5 (1.7) | 0.23 |
| **BASAL RESPIRATORY MEASURES** | | |  |  |  |  |  |  |  |  |  |  |
| FVC(L/sec) | 2.1  [1.7-2.6] | 2.0  [1.6-2.6] | 2.0  [1.7-2.7] | 0.71 | 2.0  [1.6-2.6] | 2.1  [1.7-2.6] | 2.1  [1.7-2.6] | 0.82 | 1.9  [1.5-2.4] | 2.0  [1.7-2.6] | 2.2  [1.6-2.6] | 0.46 |
| FEV_1_(L/sec) | 1.7  [1.4-2.1] | 1.7  [1.4-2.2] | 1.7  [1.6-2.3] | 0.38 | 1.7  [1.3-2.3] | 1.7  [1.4-2.1] | 1.6  [1.4-2.1] | 0.42 | 1.6  [1.3-1.9] | 1.7  [1.4-2.1] | 1.7  [1.4-2.1] | 0.14 |
| FEF_2575_ | 1.8  [1.3-2.4] | 1.7  [1.3-2.3] | 2.1  [1.6-2.4] | 0.07 | 1.9  [1.3-2.8] | 1.7  [1.4-2.1] | 1.8  [1.2-2.4] | 0.43 | 1.8  [1.2-2.3] | 1.7  [1.3-2.4] | 1.8  [1.3-2.5] | 0.97 |
| IL-8 (pg/ml) | 202.7  [92.7-391.0] | 196.6  [86.0-603.0] | 188.9  [74.3-470.9] | 0.99 | 147.6  [76.0-471.9] | 231.2  [112.0-603.0] | 139.6  [55.0-436.6] | 0.31 | 181.0  [55.0-549.0] | 194.6  [101.6-824.0] | 232.0  [131.6-397] | 0.97 |

*N=257; † N=199; ‡χ^2^  test (mean(SD))

§Wilcoxon-Mann-Whitney test (median [Q25-Q75]}; ll ANOVA test (Mean (SD)) ;

Table S3. Air pollution levels during the study from the Mexico City monitoring network, 1998-2004

|  | Mean | SD | Min | Max | Q_25_ | Q_75_ | Median  (IQR) |
| --- | --- | --- | --- | --- | --- | --- | --- |
| Ozone (ppb) |  |  |  |  |  |  |  |
| 24h average | 29.5 | 11.4 | 5.9 | 80.4 | 20.6 | 37.1 | 28.7 (16.5) |
| 8h moving average (max) | 57.6 | 31.8 | 8.0 | 184.0 | 31.3 | 80.1 | 52.1 (48.8) |
| 1h maximum | 96.9 | 44.2 | 10.0 | 309.0 | 64.0 | 124.0 | 95.0 (60.0) |
| Temperature minimum (°C) | 10.5 | 3.4 | 0.7 | 18.8 | 8.2 | 13.2 | 11.2 (5.0) |

Table S4. Effect of ozone on FEF_25-75_ (per 1-hr 60 ppb on the day prior to spirometric test) according to genotype

|  | **FEF_25-75_(ml/s)*** | | | | | |
| --- | --- | --- | --- | --- | --- | --- |
| Genotype | All asthmatics | | | | Persistent asthmatics | |
|  | n | Coeff (95%CI) | | | n | Coeff (95%CI) |
| **Overall effect of ozone** | 257 | -3.3 (-24.5, 17.9) | | | 164 | -16.2 (-42.1, 9.7) |
| GSTM1, # copies |  |  | | |  |  |
| 0 (null) | 93 | | -25.8 (-58.7, 7.1) | 65 | | -36.6 (-73.1, -0.1) **€** |
| 1 | 129 | | 3.9 (-25.6, 33.2) | 81 | | -15.0 (-51.8, 21.8) |
| 2 | 35 | | 22.2 (-39.0, 83.4) | 18 | | 23.7 (-60.9,108.4) |
| 1+ 2 (positive) | 164 | | 9.2 (-17.3, 35.6) | 99 | | -5.4 (-39.5, 28.7) |
| GSTP1 rs1695 |  | |  |  | |  |
| Val/Val | 85 | | -6.6 (-44.2, 31.0) | 54 | | -23.1 (-69.4, 23.2) |
| Ile/Val | 113 | | -7.2 (-35.4, 21.0) | 72 | | -6.8 (-43.3, 29.6) |
| Ile/Ile | 59 | | 9.0 (-46.3, 64.3) | 38 | | -24.6 (-85.8,36.6) |
| Ile/Val+Ile/Ile | 172 | | -2.1 (-28.0, 23.8) | 110 | | -12.6 (-44.5, 19.2) |
| **NQO1 rs1800566** § |  | |  |  | |  |
| Pro/Pro | 67 | | -16.6 (-60.4, 27.2) | 46 | | -35.0 (-85.8, 15.8) |
| Pro/Ser | 88 | | -6.1 (38.2, 26.0) | 61 | | -12.6 (-51.4, 26.2) |
| Ser/Ser | 44 | | 17.8 (-31.9, 67.6) | 30 | | 6.0 (-56.3, 68.3) |
| Pro/Ser+Ser/Ser | 132 | | 3.4 (-23.7, 30.4) | 91 | | -4.2 (-37.1, 28.7) |

*Models were adjusted for gender, age, BMI, height, time, cohort, use of bronchodilator, and minimum temperature; All asthmatics: n=257 and 4548 repeated measures; persistent (mild, moderate or severe) asthmatics: n=164 and 3029 repeated measures

§ NQO1 : all asthmatics n=199 and 3842 repeated measures; persistent (mild, moderate or severe): n=137 and 2715 repeated measures.

**€** p≤0.05Table S5. Effect of ozone on FEF_25-75_ (per 1-hr 60 ppb on the day prior to spirometric test) according to vitamin C intake

|  | FEF_25-75_(ml/s)* | | | |
| --- | --- | --- | --- | --- |
| Vitamin C intake | All asthmatics | | Persistent asthmatics | |
|  | n | Coeff (95%CI) | n | Coeff (95%CI) |
| 30 – 105 mg/day | 85 | -31.2 (-71.2, 8.8) | 45 | -61.2 (-114.0, -8.4) **€** |
| >105- 226 mg/day | 94 | 6.2 (-26.7, 39.2) | 60 | -17.6 (-57.6, 22.3) |
| 280- 447 mg/day | 78 | 3.2 (-31.8, 38.3) | 59 | 5.9 (-37.6, 49.5) |

***** Models were adjusted for gender, age, BMI, height, time, cohort, use of bronchodilator, and minimum temperature; All asthmatics: n=257 and 4548 repeated measures; persistent (mild, moderate or severe) asthmatics: n=164 and 3029 repeated measures.

**€** p≤0.05
